# Hypersensitivity of Vagal Pulmonary Afferents Induced by Tumor Necrosis Factor Alpha in Mice

**DOI:** 10.3389/fphys.2017.00411

**Published:** 2017-06-14

**Authors:** Ruei-Lung Lin, Qihai Gu, Lu-Yuan Lee

**Affiliations:** ^1^Department of Physiology, University of KentuckyLexington, KY, United States; ^2^Department of Biomedical Sciences, Mercer UniversityMacon, GA, United States

**Keywords:** airway, lung, C-fiber, rapidly adapting receptor, cytokine, inflammation, asthma

## Abstract

Tumor necrosis factor alpha (TNFα), a pro-inflammatory cytokine, plays a significant role in the pathogenesis of allergic asthma. Inhalation of TNFα also induces airway hyperresponsiveness in healthy human subjects, and the underlying mechanism is not fully understood. A recent study reported that TNFα caused airway inflammation and a sustained elevation of pulmonary chemoreflex responses in mice, suggesting a possible involvement of heightened sensitivity of vagal pulmonary C-fibers. To investigate this possibility, the present study aimed to investigate the effect of a pretreatment with TNFα on the sensitivity of vagal pulmonary afferents in anesthetized mice. After TNFα (10 μg/ml, 0.03 ml) and vehicle (Veh; phosphate buffered saline (PBS), 0.03 ml) were administered by intra-tracheal instillation in each mouse of treated (TNF) and control (Veh) groups, respectively, the peak activity of pulmonary C-fibers in response to an intravenous bolus injection of a low dose of capsaicin (Cap; 0.5 μg/kg) was significantly elevated in TNF group (6.5 ± 1.3 impulses/s, *n* = 12) 24–48 h later, compared to that in Veh group (2.2 ± 0.5 impulses/s, *n* = 11; *P* < 0.05). Interestingly, the same low dose of Cap injection also evoked a distinct burst of discharge (2.4 ± 0.7 impulses/s) in 75% of the silent rapidly adapting receptors (RARs), a subtype of RARs exhibiting no phasic activity, in TNF group, but did not stimulate any of the silent RARs in Veh group. To further determine if this sensitizing effect involves a direct action of TNFα on these sensory nerves, the change in intracellular Ca^2+^ concentration in response to Cap challenge was measured in isolated mouse vagal pulmonary sensory neurons. The Cap-evoked Ca^2+^ influx was markedly enhanced in the neurons incubated with TNFα (50 ng/ml) for ~24 h, and this sensitizing effect was attenuated in the neurons isolated from the TNF-receptor double homozygous mutant mice. In conclusion, the TNFα pretreatment enhanced the Cap sensitivity in both pulmonary C-fibers and silent RARs, and the action was mediated through TNF receptors. These sensitizing effects of TNFα may contribute, at least in part, to the pathogenesis of airway hyperresponsiveness induced by this cytokine.

## Introduction

It is well-documented that tumor necrosis factor alpha (TNFα), a pro-inflammatory cytokine, plays a significant role in the pathogenesis of chronic airway inflammatory diseases such as allergic asthma (Thomas, 2001; Howarth et al., 2005; Berry et al., 2006; Heffler et al., 2007; Brightling et al., 2008). Inhalation of aerosolized TNFα can also induce bronchial hyperresponsiveness accompanied by airway inflammation in healthy human subjects (Thomas et al., 1995), but the underlying mechanism is not fully understood. One of the prominent pathophysiological features of inflammation-induced bronchial hyperresponsiveness is a heightened sensitivity of airway sensory nerves (Lee and Yu, 2014; Mazzone and Undem, 2016). A recent study carried out in our laboratory has demonstrated that intra-tracheal instillation of TNFα 24 h earlier caused airway inflammation and a sustained (>48 h) elevation of pulmonary chemoreflex sensitivity in mice (Lin et al., 2013). Because pulmonary chemoreflexes are known to be elicited by stimulation of vagal bronchopulmonary C-fiber afferents (Lee and Pisarri, 2001), this finding suggested a possible involvement of these vagal afferents (Lin et al., 2013). However, whether the sensitivity of these afferents is actually elevated following the TNFα treatment in intact animals remains to be determined.

Previous investigators have shown that the TNFα treatment induced a sensitizing effect on the nociceptive neurons in dorsal root ganglia (DRG), the counterpart of pulmonary C-fiber neurons innervating other organs, and contributed to the development of lingering inflammatory pain in somatic tissues (Cunha et al., 1992; Nicol et al., 1997). It has been suggested that this hyperalgesic effect was mediated through an action on the TNF receptors, TNFR1 and TNFR2, on the cell surface, and an increase in the sensitivity and/or expression of transient receptor potential vanilloid type 1 (TRPV1) receptors in DRG neurons (Heffler et al., 2007). Whether the same mechanism is involved in the TNFα-induced hypersensitivity of vagal pulmonary afferents is not yet known.

Among the three major types of vagal bronchopulmonary afferents, C-fiber represents the majority (Jammes et al., 1982) and plays an important role in the regulation of cardiopulmonary function in both healthy and disease conditions (Coleridge and Coleridge, 1984; Lee and Pisarri, 2001). Bronchopulmonary C-fibers can be activated by various endogenous inflammatory mediators and inhaled chemical irritants; stimulation of these afferents elicits an array of powerful reflex responses including cough, bronchoconstriction, and airway hypersecretion (Coleridge and Coleridge, 1984; Lee and Pisarri, 2001; Lee and Yu, 2014); intense and/or sustained stimulation can lead to neurogenic inflammation (Baluk et al., 1992; De Swert and Joos, 2006). Thus, when the sensitivity of these afferents is elevated, a mild stimulus may trigger exaggerated reflex responses, and contribute to the airway hyperresponsiveness. One of the characteristic traits of these C-fiber sensory neurons is an abundant expression of the TRPV1 channel, a polymodal transducer involved in the manifestation of various symptoms of airway hypersensitivity (Geppetti et al., 2006; Lee and Gu, 2009). The other two major types of vagal bronchopulmonary sensory receptors, rapidly adapting receptors (RARs) and slowly adapting receptors (SARs), are myelinated afferents and function primarily as mechanoreceptors (Lee and Yu, 2014). However, an unexpected expression of TRPV1 in RARs and SARs resulting from chronic allergic airway inflammation has been reported in rats (Zhang et al., 2008). Whether this may occur in the TNFα-induced airway inflammation in mice is not known.

To answer these questions, the first study series was designed to determine the effect of intra-tracheal administration of TNFα into the lung on the sensitivity of vagal bronchopulmonary afferents to capsaicin (Cap), a selective TRPV1 agonist (Caterina et al., 1997; Nilius et al., 2007), in anesthetized mice 24–48 h later, using the single-fiber recording technique. Because of the diverse actions of TNFα on a number of other target cells in the airways including neutrophils, eosinophils, and endothelial cells (Thomas, 2001), it was impossible to determine if the sensitizing effect is mediated through a direct action of TNFα on these neurons in a whole-animal preparation. Therefore, in the second study series the effect of TNFα was determined in isolated mouse pulmonary sensory neurons using the Ca^2+^ imaging technique. A possible involvement of TNF receptors was further investigated in mice in which both types of TNF receptors were mutated.

## Methods and materials

This study consisted of both *in vivo* and *in vitro* experiments. The experimental procedures described below were in accordance with the recommendation in *Guide for the Care and Use of Laboratory Animals* published by the National Institutes of Health, and also approved by the University of Kentucky Institutional Animal Care and Use Committee.

### *In-vivo* study

#### Pretreatment with TNFα

Young male C57BL6/J mice (12-wk old) were anesthetized by inhalation of vaporized isoflurane (2% in O_2_) via a nosecone. After a small (~2 mm) mid-line incision was made on the ventral neck skin to expose the trachea, a small volume (0.03 ml) of TNFα (10 μg/ml) or phosphate buffered saline (PBS) was instilled into the trachea via a 28-gauge needle, and the incision was then closed by tissue adhesive (Vetbond, 3M, St. Paul, MN, USA). This dose of TNFα was chosen based upon our pilot experiments performed in an earlier study (Lin et al., 2013) to determine the minimal dose required to induce airway hypersensitivity in mice. Morton and coworkers have recently reported a similar dose of TNFα to be effective in generating airway inflammation in mice (Morton et al., 2016). Experiments were carried out 24–48 h later.

#### Animal preparation

Mice were initially anesthetized with an intraperitoneal injection of α-chloralose (70 μg/g) and urethane (1 mg/g) dissolved in a 2% borax solution; supplemental doses (one-tenth of the initial dose) of the same anesthetics were injected intravenously (iv) to maintain abolition of pain reflexes induced by tail-pinch. For administration of pharmacological agent (s), a catheter was inserted into the left jugular vein and advanced until its tip was positioned just above the right atrium. A catheter was inserted into femoral artery and connected to a pressure transducer (P23AA, Statham, Hato Rey, Puerto Rico) for recording the arterial blood pressure (ABP) and heart rate (HR). A short tracheal cannula was inserted just below the larynx via a tracheotomy. Tracheal pressure (P_T_) was continuously recorded (MP45-28, Validyne, Northridge, CA, USA) via a side-port of the tracheal cannula. After a midline thoracotomy was performed, the lung was artificially ventilated with a respirator (Model 845, Hugo Sachs Elektronik, March, Germany); the expiratory outlet of the respirator was placed under 3-cmH_2_O pressure to maintain a stable and near-normal functional residual capacity. Tidal volume (V_T_) and respiratory rate were set at 8 μl/g and 180 breaths/min, respectively, to mimic those of anesthetized mice. Body temperature was maintained at ~36°C by means of heating pad placed under the animal lying in a supine position.

#### Electrophysiological recording of vagal bronchopulmonary afferent activity

Single-unit activities of vagal bronchopulmonary afferents were recorded following the protocol modified from our previous studies in rats (Ho et al., 2001). Briefly, the right cervical vagus nerve was sectioned, and its caudal end was placed on a small dissecting platform and immersed in a pool of mineral oil. A thin filament was teased away from the desheathed nerve trunk and placed on a platinum-iridium hook electrode. Action potentials were amplified by a preamplifier (Model P511K, Grass Technologies, Warwick, RI, USA), and monitored by an audio monitor (Model AM8RS, Grass Technologies, Warwick, RI, USA). The thin filament was further split until the afferent activity arising from a single unit was electrically isolated.

**Figure 1:**
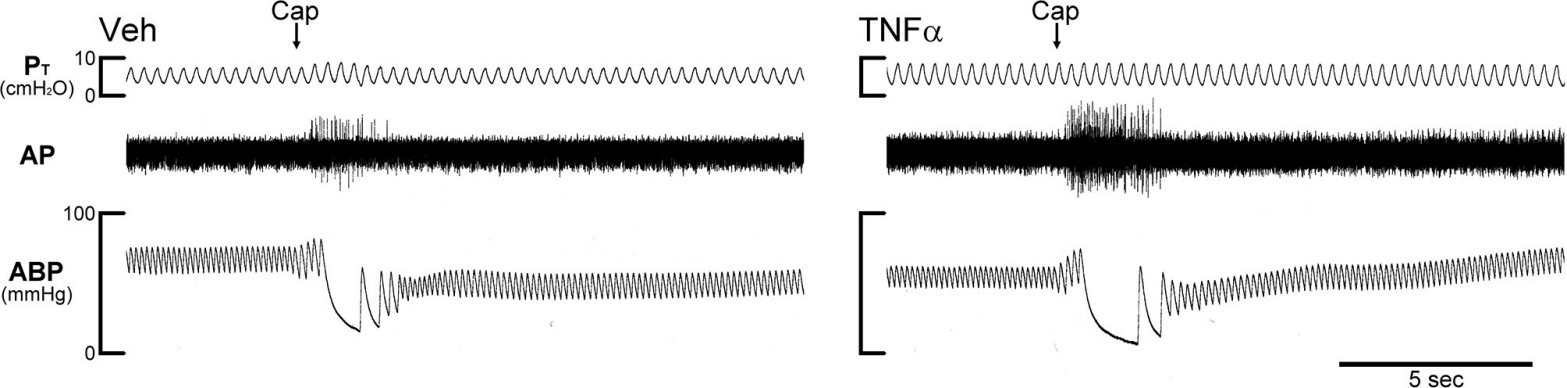
Experimental records illustrating the effect of TNFα on the responses of pulmonary C-fibers to capsaicin in anesthetized, open-chest and artificially ventilated mice. Vehicle (Veh; 0.03 ml of PBS) and TNFα (10 μg/ml, 0.03 ml) were administered by intra-tracheal instillation into the lungs of Veh and TNFα mice, respectively, ~24 h earlier. Arrows were added to mark the time when capsaicin (Cap; 1.0 μg/kg) was injected intravenously as a bolus. Both receptor locations were in the right lung. Body weights of Veh and TNFα mice were 27.8 and 25.9 g, respectively. P_T_, tracheal pressure; AP, action potential; ABP, arterial blood pressure.

The afferent activity of a pulmonary C-fiber was first searched by hyperinflation of the lung (*P*_T_ > 30 cmH_2_O) and then identified by the immediate (delay < 1 s) response to a bolus iv injection of Cap (1 μg/kg); an example is shown in Figure 1. RARs and SARs were searched initially by their responses to hyperinflation of the lung, and then further identified by their adaptation indexes (AIs) in response to lung inflation; AI was calculated in each fiber by dividing the difference in fiber activity (FA) between the first 2 s during a constant-pressure lung hyperinflation (*P*_T_ = 30 cmH_2_O; e.g., **Figure 3**) by the FA of the first second, and expressed as a percentage (Widdicombe, 1954). Fibers with AIs of <80 and >80% were classified as SARs and RARs, respectively.

The activity of each sensory nerve was recorded when low and high doses of iv injections of Cap (0.5–1.0 μg/kg) were administered with 15–20 min elapsed between two injections. The FA signal was recorded continuously at a sampling rate of 3–20 kHz and analyzed by a computer and a data acquisition system (Biocybernetics TS-100) for 20 s before and 60 s after each injection. Baseline FA was averaged over the 10-s period immediately preceding the Cap injection, and the peak response was defined as the highest FA averaged over 2-s duration (or 6 breaths) within the first 5 s after the injection.

The general locations of these sensory receptors in the lung structure were identified by their responses to the gentle pressing of the outer surface of the lung with a blunt-ended glass rod at the end of each experiment. Animals were then euthanized by iv injection of 3-M KCl (0.2 ml).

### *In-vitro* study

The experiments were carried out in two groups of young male mice (8–12 week old): 1) wild-type (WT; B6129SF2/J); 2) TNF-receptor double homozygous mutant mice (TNF^−/−^; 129S-*Tnfrsf1a*^*tm*1*Imx*^*Tnfrsf1b*^*tm*1*Imx*^/J) in which both types of TNF receptors, TNFR1 and TNFR2, were mutated. All mice were purchased from the Jackson Laboratory (Bar Harbor, ME, USA).

#### Fluorescent labeling and isolation of vagal pulmonary sensory neurons

Sensory neurons innervating the lungs and airways were identified by retrograde labeling from the lungs by using the fluorescent tracer 1,1′-Dioctadecyl-3,3,3′,3′-tetramethylindocarbocyanine perchlorate (DiI, Sigma-Aldrich, St. Louis, MO, USA; Kwong and Lee, 2002). After a small midline incision was made on the ventral neck skin of the mouse during anesthesia with inhalation of vaporized isoflurane (2% in O_2_), DiI (0.15 mg/ml; 0.025 ml) was instilled into the mouse trachea and lung via a needle (28 gauge). The incision was then closed (Vetbond, 3M, St. Paul, MN, USA). Seven to ten days later, DiI-pretreated animals were anesthetized and decapitated. The head was immediately immersed in ice-cold DMEM/F-12 solution followed by quick extraction of the jugular-nodose ganglia complex; as described by Nassenstein et al. (2010), the mouse jugular and nodose ganglia were fused together in a single structure, and could not be separated as in larger rodents (e.g., guinea pig, rat). Each ganglion was then desheathed, cut in smaller pieces, and placed in a mixture of type IV collagenase (0.04%) and dispase II (0.02%), and incubated for 80 min in 5% CO_2_ in air at 37°C. After digestion, centrifugation and re-suspension, cells were dissociated by gentle trituration with small-bore, fire-polished Pasteur pipettes. Myelin debris was discarded after centrifugation of the dispersed cell suspension (500 G, 8 min). The cell pellets were resuspended in the modified DMEM/F-12 solution, plated onto poly-l-lysine-coated glass coverslips, and then incubated overnight (5% CO_2_ in air at 37°C).

#### Measurement of Ca^2+^ transient in isolated mouse pulmonary sensory neurons

Changes in the intracellular Ca^2+^ concentration, [Ca^2+^]_i_, in isolated pulmonary sensory neurons were measured by the ratiometric method as reported in our previously study (Gu et al., 2003). Briefly, cultured cells (as described above) were washed and maintained in an extracellular solution (ECS). Ca^2+^ transients were measured in these cells with a digital fluorescence microscope (Axiovert 100, Carl Zeiss, Thornwood, NY, USA) equipped with a variable filter wheel (Sutter Instruments, Novato, CA, USA) and a digital CCD camera (Princeton Instruments, Trenton, NJ, USA). Cells were incubated with 5-μM Fura-2 AM (Life Technologies, Grand Island, NY, USA), a Ca^2+^ indicator, for 30 min at 37°C, rinsed (× 3) with ECS and then allowed to de-esterify for 30 min before use. Dual images (340- and 380-nm excitation, 510-nm emission) were collected, and pseudocolor ratiometric images were monitored during the experiments by using the software Axon Imaging Workbench (Axon Instruments, Union City, CA, USA; Grynkiewicz et al., 1985).

The coverslip containing the cells was mounted into a chamber continuously perfused with an ECS during the experiment by a gravity-fed valve-controlled system (VC-66CS, Warner Instruments, Hamden, CT, USA) at a constant rate of ~2 ml/min. The Fura-2 fluorescence 340/380 ratio was continuously recorded and analyzed at 2-s intervals, and was converted to [Ca^2+^]_i_ in nM by the calibration, as described in our previous study (Hu et al., 2010); the Ca^2+^ transient was measured as the difference between the peak amplitude (4-s average) after the challenge and the baseline (30-s average). A high concentration of KCl (60 mM, 20-s perfusion) was applied at the end of each experiment to determine the cell viability. Cells that did not respond to the KCl challenge were considered non-excitable, and data were not included for analysis.

Two study series were carried out: *Study 1:* to determine the effect of a pretreatment with TNFα on the responses of Ca^2+^ transient to increasing concentrations of Cap in vagal pulmonary sensory neurons; two matching dishes of pulmonary jugular-nodose ganglia neurons cultured from the same ganglion of the same mouse were incubated with vehicle (Veh group) and 50 ng/ml of TNFα (TNF group) in culture medium for 24 h, respectively, and their responses to three concentrations of Cap (30, 100 and 300 nM; 30 s application and 10 min recovery) were then determined. This range of Cap concentrations was selected based upon our previous study (Hu et al., 2010). *Study 2*: to determine if the effect observed was mediated through the TNF receptors, TNFR1 and TNFR2, expressed in vagal pulmonary sensory neurons, the experimental protocols described in *Study 1* was repeated in the pulmonary jugular-nodose neurons isolated from WT and TNF^−/−^ mice that had incubated with 50 ng/ml of TNFα in culture medium for 24 h; their responses were then compared.

### Chemical agents

All chemicals were purchased from Sigma-Aldrich (St. Louis, MO, USA), except TNFα (ProSpec-Tany TechnoGene, Rehovot, Israel), dispase II (Roche, Indianapolis, IN, USA), DMEM/F-12 (Invitrogen, Carlsbad, CA, USA), and Fura-2 AM (Life Technologies, Grand Island, NY, USA). A stock solution of TNFα (100 μg/ml) was diluted in DMEM/F-12 culture medium to the desired concentration daily. A stock solution of Cap (1 mM) was dissolved in 1% Tween 80, 1% ethanol, and 98% saline, and prepared daily by dilution with PBS and ECS in *in-vivo* and *in-vitro* experiments, respectively. The ECS contained (in mM): 5.4 KCl, 136 NaCl, 1.0 MgCl_2_, 1.8 CaCl_2_, 0.33 NaH_2_PO_4_, 10 glucose, 10 HEPES, and a pH level adjusted to 7.4 with NaOH and the osmolarity to 300 mOsm.

### Statistical analysis

Data were compared using either one-way or two-way analysis of variance (ANOVA), and pair-wise comparisons were made with a *post hoc* analysis (Fisher's least significant difference). *P* < 0.05 was considered significant. All data are reported as means ± SEM.

## Results

### *In-vivo* study

A total of 125 vagal bronchopulmonary afferents were studied in 40 anesthetized, open-chest mice: 17 C-fibers, 37 RARs (24 silent and 13 phasic) and 13 SARs in the Veh group (23 mice pre-treated with PBS); and 14 C-fibers, 32 RARs (22 silent and 10 phasic) and 12 SARs in the TNF group (17 mice pretreated with TNFα). Both TNFα and Veh were administered 24–48 h before the experiments.

**Figure 2:**
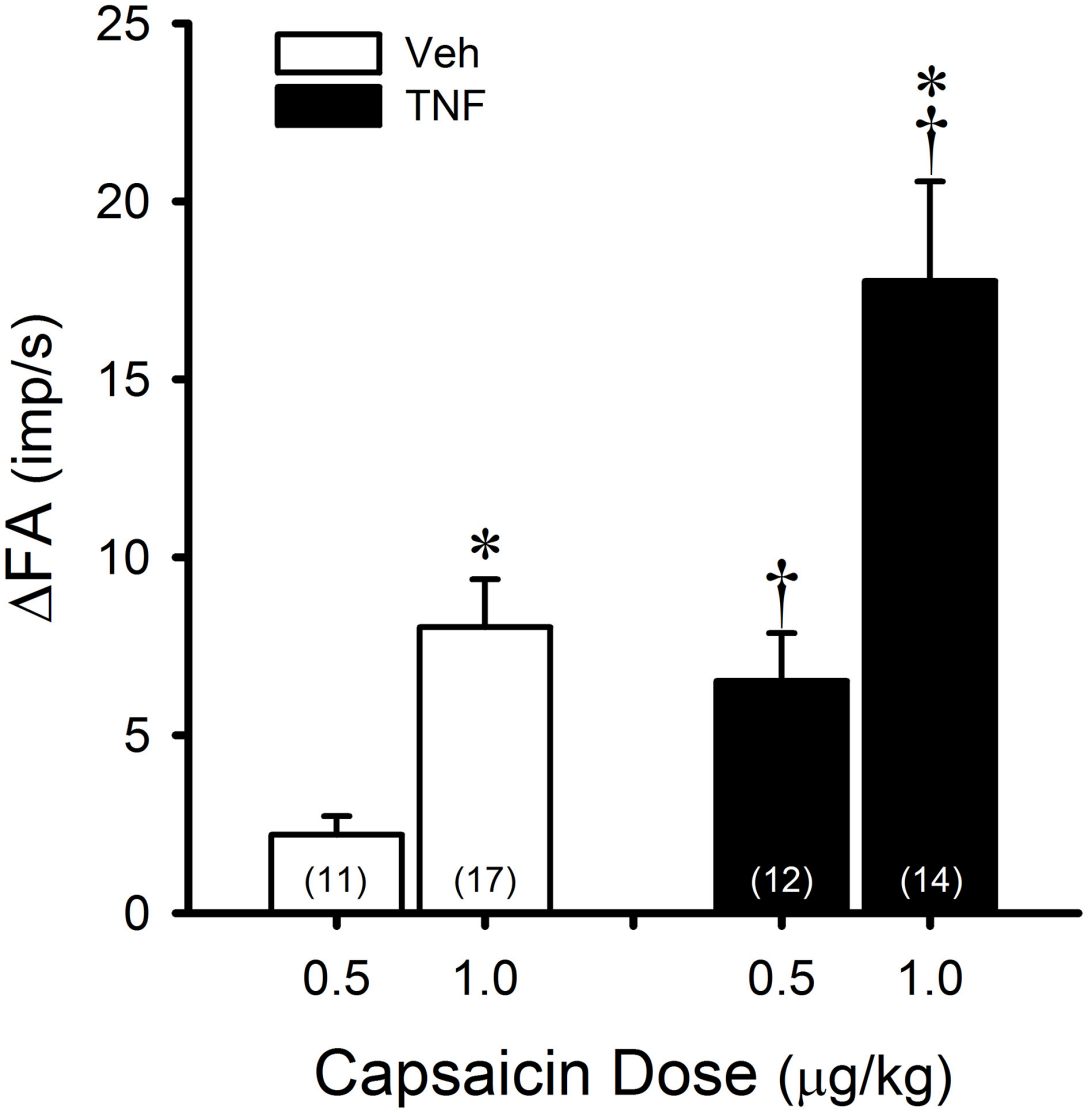
TNFα enhances capsaicin (Cap) sensitivity in pulmonary C-fibers. Veh (open bars): responses to intravenous bolus injections of Cap 24–48 h after instillation of vehicle (0.03 ml of PBS). TNF (closed bars): responses to same doses of Cap 24–48 h after instillation of TNFα (10 μg/ml, 0.03 ml). ΔFA: the increase in fiber activity (FA; impulses/s) from baseline (averaged over the 10-s period immediately preceding the injection) to the peak response (averaged over 2-s duration) within the first 5 s after the injection. The number in each bar represents the number of fibers studied under that condition. *Significantly different from the low dose of Cap (0.5 μg/kg; *P* < 0.05). ^†^Significantly different from the Veh group (*P* < 0.05). Data are means ± SEM.

In Veh-treated mice, an intravenous bolus injection of Cap triggered an abrupt (latency < 1 s) and short-duration (3–5 s) of discharge in pulmonary C-fibers (e.g., Figure 1) accompanied by bradycardia and hypotension, resembling the pulmonary chemoreflex responses, which were probably elicited by activation of pulmonary C-fibers conducted by the intact left vagus nerve. The afferent responses of pulmonary C-fibers to Cap injections were dose-dependent (Figure 2). In TNFα-treated mice, the pulmonary C-fiber responses to both low and high doses of Cap were significantly higher than that in Veh-treated mice: the ΔFA evoked by the low dose of Cap (0.5 μg/kg) was 2.2 ± 0.5 impulses/s (imp/s; *n* = 11) in the Veh group, and 6.5 ± 1.3 imp/s (*n* = 12; *P* < 0.05) in the TNF group; the ΔFA evoked by the high dose of Cap (1.0 μg/kg) was 8.1 ± 1.4 imp/s (*n* = 17) in the Veh group, and 17.8 ± 2.8 imp/s (*n* = 14; *P* < 0.01) in the TNF group. The Cap injection (1.0 μg/kg) increased P_T_ in both TNF and Veh groups, but the ΔP_T_ evoked by Cap was not significantly different between the two groups (*P* > 0.05).

In Veh-treated (control) mice, pulmonary C-fibers are relatively insensitive to lung inflation: in general, they were either not or only weakly stimulated by hyperinflation of the lung (*P*_T_ = 30 cmH_2_O). This weak response of pulmonary C-fibers to lung inflation was not altered by the TNFα pretreatment.

**Figure 3:**
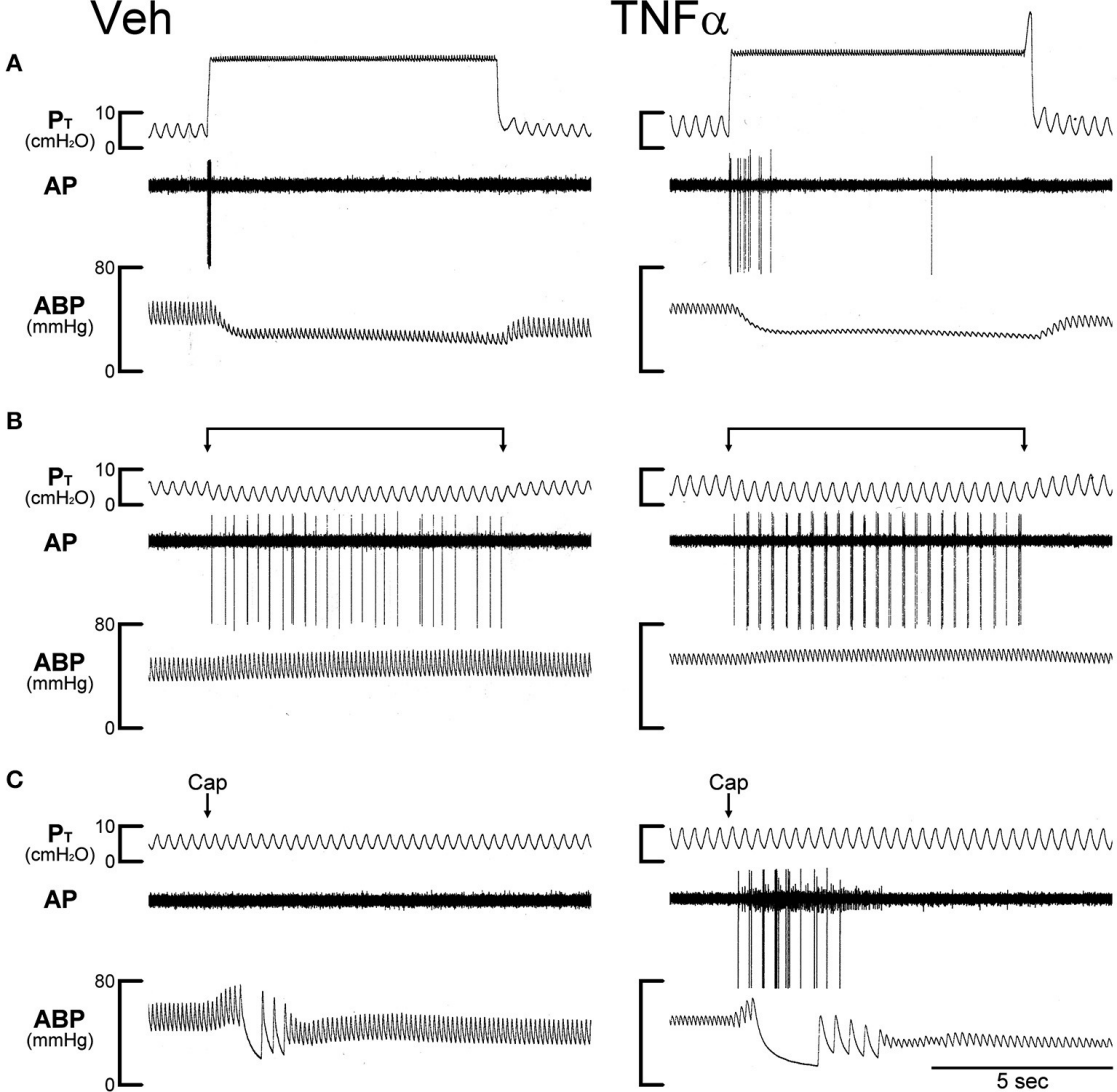
Experimental records illustrating the effect of TNFα on the responses of silent RARs to capsaicin, inflation and deflation of the lung in two anesthetized, open-chest and artificially ventilated mice. Veh (0.03 ml of PBS) and TNFα (10 μg/ml, 0.03 ml) were administered ~24 h earlier by intra-tracheal instillation into the lungs of Veh and TNFα mice, respectively. **(A)** Responses to constant pressure (30 cmH_2_O) hyperinflations of the lungs for 8 s when the ventilator was turned off. **(B)** Responses to lung deflations when the expiratory line of the ventilator was exposed to atmospheric pressure. **(C)** Responses to intravenous bolus injections of capsaicin (1.0 μg/kg) at arrows. Both receptor locations were in the right lung. Body weights of Veh and TNFα mice were 31.8 and 25.9 g, respectively. Notice that in the TNFα-pretreated mouse in **(C)** a pulmonary C-fiber (with a smaller amplitude of AP) was also stimulated by Cap injection, but was not activated by either lung inflation or deflation. P_T_, tracheal pressure; AP, action potential; ABP, arterial blood pressure.

**Figure 4:**
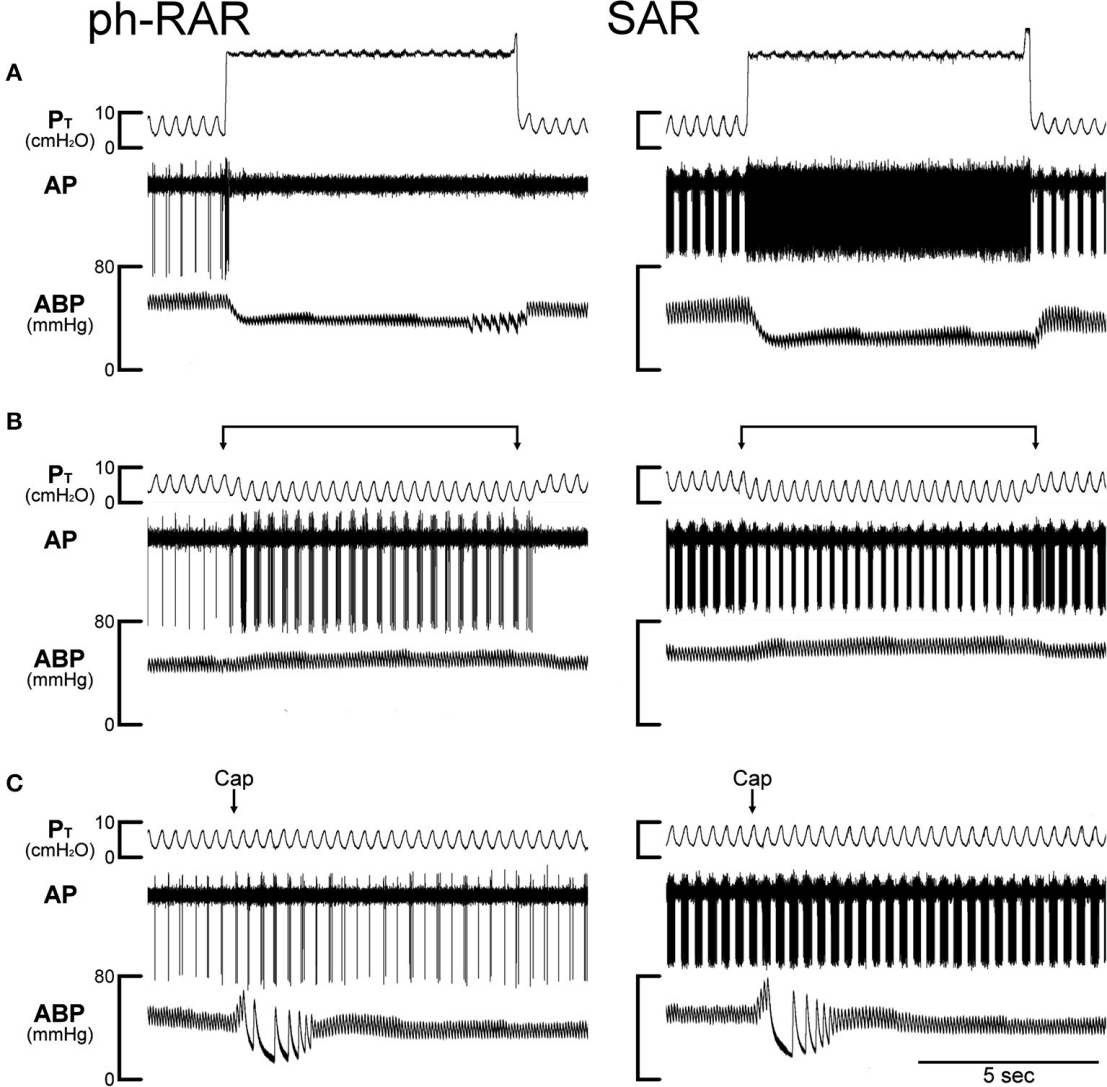
Experimental records illustrating the effect of TNFα on the responses of a phasic rapidly adapting receptor (ph-RAR) and a slowly adapting receptor (SAR) to capsaicin, inflation and deflation of the lung in an anesthetized, open-chest and artificially ventilated mouse. TNFα (10 μg/ml, 0.03 ml) was administered by intra-tracheal instillation into the lungs of the mouse ~24 h earlier. **(A)** Responses to constant pressure (30 cmH_2_O) hyperinflations of the lungs for 8 s when the ventilator was turned off. **(B)** Responses to lung deflations when the expiratory line of the ventilator was exposed to atmospheric pressure. **(C)** Responses to intravenous bolus injections of capsaicin (1.0 μg/kg) at arrows. Both receptor locations were in the right lung. Body weight of the mouse was 24.2 g. P_T_, tracheal pressure; AP, action potential; ABP, arterial blood pressure.

RARs exhibited a rapid adaptation to constant-pressure (*P*_T_ = 30 cmH_2_O maintained for 8 s) lung inflation: AI > 80% (e.g., Figure 3). They were also activated by lung deflation when the expiratory line of the respirator was exposed to atmospheric pressure for 8 s (end-expiratory *P*_T_ = 0 cmH_2_O; e.g., Figures 3, 4). A large percentage of the RARs exhibited distinct phasic baseline activity that was synchronous with either expiratory or inspiratory phase of respiratory cycles (e.g., Figure 4). The remaining RARs had no detectable or very little respiratory-related baseline activity (e.g., Figure 3). These two types of RARs were categorized as phasic RARs and silent RARs in this study.

**Figure 5:**
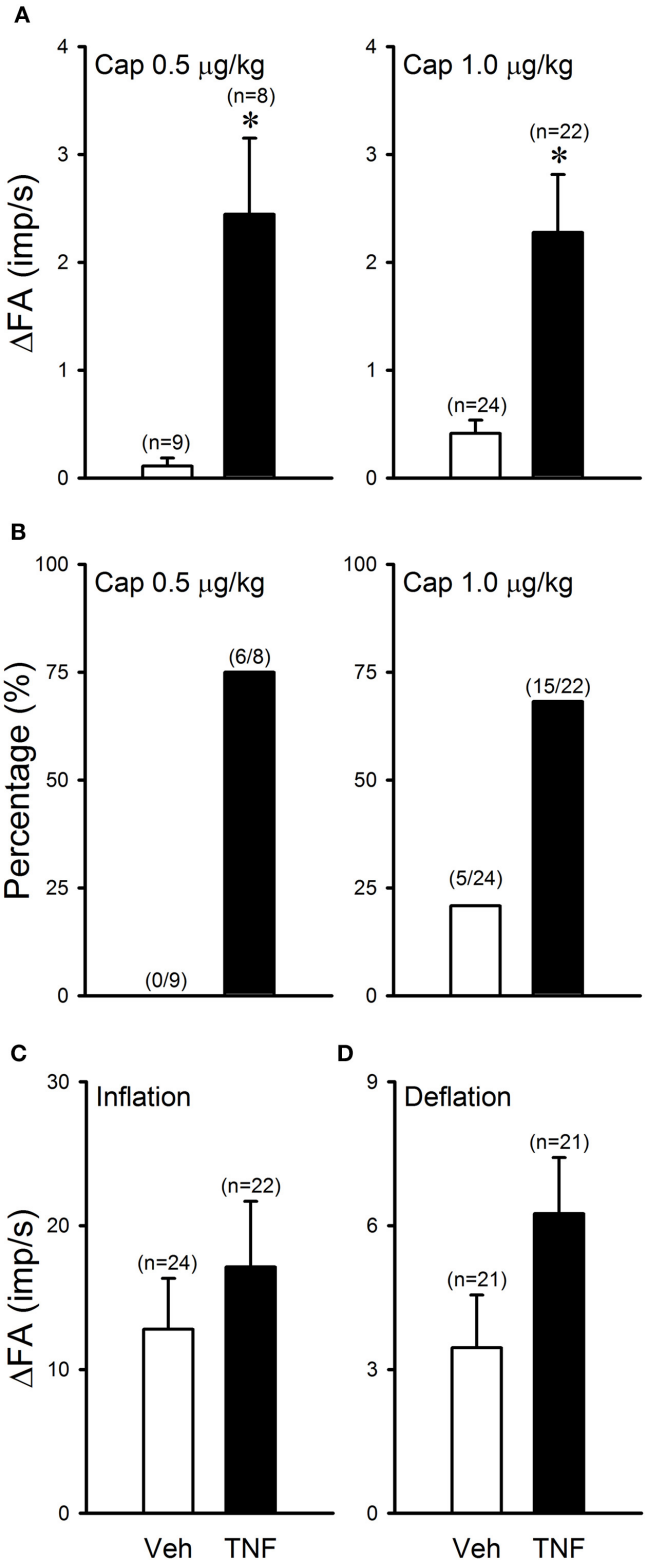
TNFα enhances capsaicin sensitivity in silent RARs: a comparison between Veh (open bars) and TNF group (closed bars). **(A)** Responses to Cap injections (0.5–1.0 μg/kg); ΔFA: the increase in fiber activity (FA; impulses/s) from baseline (averaged over the 10-s period immediately preceding the injection) to the peak response (averaged over 2-s duration) within the first 5 s after the injection. **(B)** Percentages of silent RARs that exhibited Cap sensitivity (ΔFA > 1.0 imp/s after Cap injection). **(C)** Response to lung hyperinflation (*P*_T_ = 30 cmH_2_O); ΔFA: the difference in FA between the FA during the first second of lung inflation and the baseline (averaged over the 10-s period preceding inflation). **(D)** Response to lung deflation; ΔFA: the difference between the FA averaged over the 8-s duration of deflation and the baseline (averaged over the 10-s period preceding the deflation). ^*^Significantly different from the Veh group (*P* < 0.05).

In Veh-treated (control) mice, injections of Cap even at high dose (1.0 μg/kg, iv) did not cause significant stimulatory effect on silent RARs (e.g., Figure 3). However, the responses of these silent RARs to Cap injections were significantly elevated in TNFα-treated mice: the ΔFA evoked by the low dose of Cap (0.5 μg/kg) was 0.1 ± 0.1 imp/s (*n* = 9) in the Veh group, and 2.4 ± 0.7 imp/s (*n* = 8; *P* < 0.01) in the TNF group (Figure 5A); the ΔFA evoked by the high dose of Cap (1.0 μg/kg) was 0.4 ± 0.1 imp/s (*n* = 24) in the Veh group, and 2.3 ± 0.5 imp/s (*n* = 22; *P* < 0.01) in the TNF group (Figure 5A). Furthermore, the number of silent RARs that were activated by Cap injections, judged by the criterion of ΔFA > 1.0 imp/s, was distinctly higher in TNFα-treated mice: e.g., none of the 9 silent RARs tested were activated by the low dose of Cap (0.5 μg/kg) in the Veh group, whereas 6 of the 8 silent RARs were stimulated by the same Cap injection in the TNF group (Figure 5B). However, there was no significant difference in the responses of silent RARs to either lung inflation (*P* > 0.05) or deflation (*P* > 0.05) between Veh- and TNFα-treated mice (Figures 5C,D).

In comparison, the TNFα pretreatment did not cause any potentiating effect on the FA responses to the same Cap injection in phasic RARs: for example, the peak FA after the injection of high dose of Cap (1.0 μg/kg) was 13.2 ± 4.2 imp/s (*n* = 13) in the Veh group, and 11.9 ± 2.2 imp/s (*n* = 10; *P* > 0.5) in the TNF group.

All of the SARs tested in this study exhibited distinct phasic baseline activity that reached a peak during either inspiratory or expiratory phase of the respiratory cycles (e.g., Figure 4); the latter was found in a relatively smaller number of fibers. The discharge of SARs showed smaller adaptation (AI < 80%) than RARs during the constant-pressure lung inflation, and their phasic activity was markedly lowered (e.g., Figure 4) or ceased completely during lung deflation. The TNFα pretreatment did not cause any potentiating effect on the FA responses to Cap injection in SARs: for example, the peak FA after the injection of high dose of Cap (1.0 μg/kg) was 58.8 ± 8.0 imp/s (*n* = 13) in the Veh group, and 43.7 ± 12.3 imp/s (*n* = 12; *P* > 0.3) in the TNF group. There was no significant difference in the response of SARs to either lung inflation (*P* > 0.05) or deflation (*P* > 0.05) between Veh and TNF groups.

### *In-vitro* study

**Figure 6:**
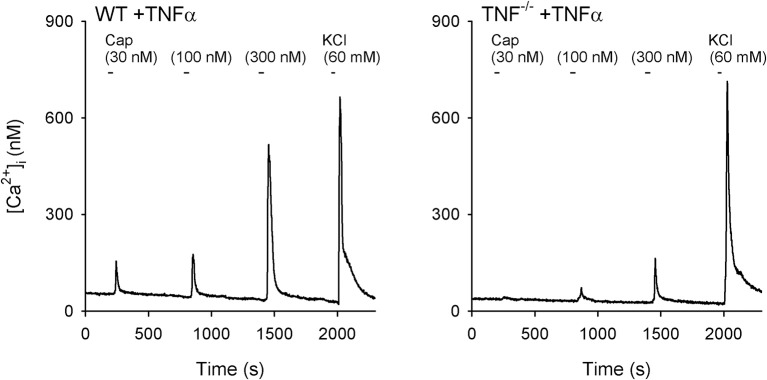
Representative experimental records illustrating the change in [Ca^2+^]_i_ evoked by increasing concentrations of capsaicin (Cap) in vagal pulmonary sensory neurons. **Left panel**: a pulmonary neuron (diameter: 24 μm) isolated from a wild-type (WT) mouse; **Right panel**: a pulmonary neuron (diameter: 22 μm) isolated from a TNF-receptor double homozygous mutant (TNF^−/−^) mouse. Both WT and TNF^−/−^ neurons had been incubated with TNFα (50 ng/ml in culture medium) for ~24 h. Cap was applied for 30 s each, and KCl solution (60 mM, 20 s) was applied to test cell vitality at the end of each experiment.

**Figure 7:**
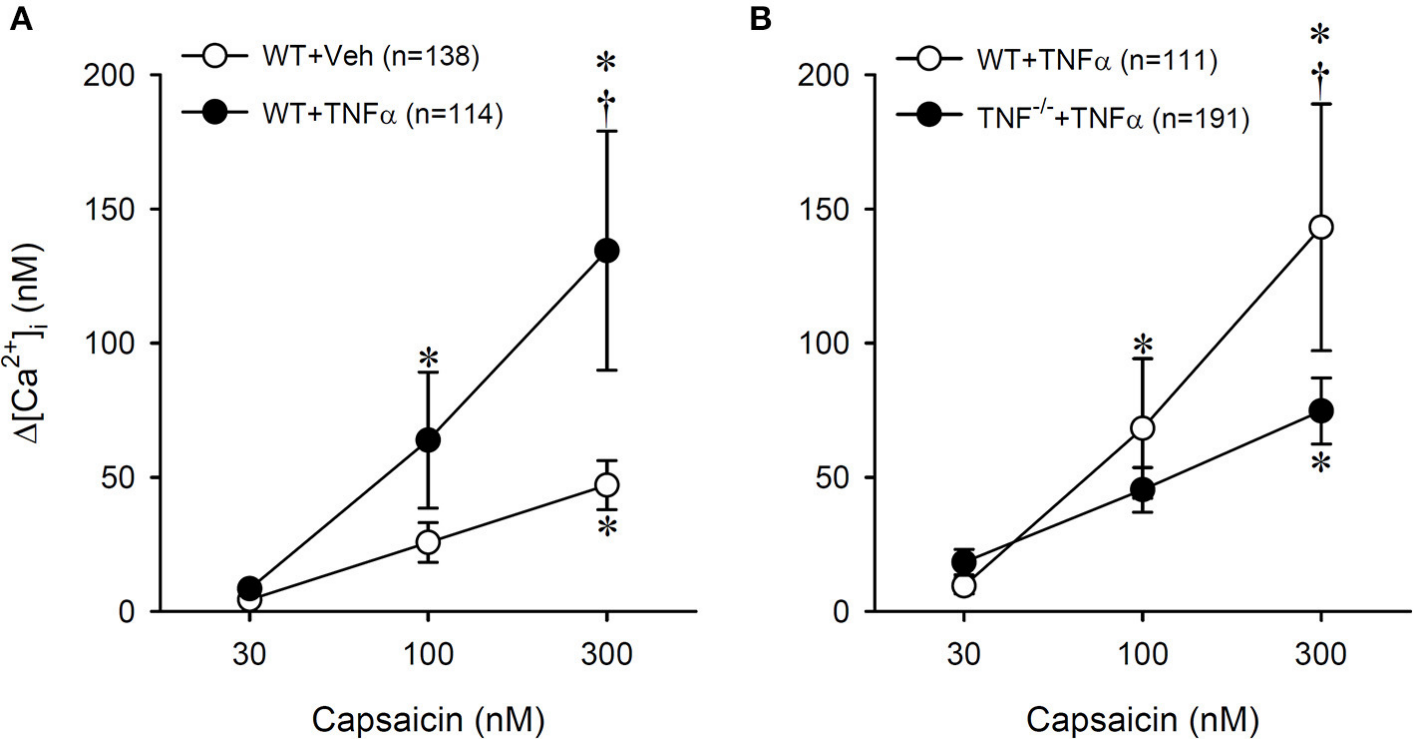
Potentiating effect of TNFα (50 ng/ml, ~24 h) on Cap-evoked calcium transient in pulmonary sensory neurons isolated from WT and TNF^−/−^ mice. **(A)** A comparison of the responses of [Ca^2+^]_i_ to increasing concentrations of Cap between Veh- and TNFα-treated neurons isolated from wild-type (WT) mice. ^*^Significantly different from the corresponding response to 30 nM capsaicin (*P* < 0.05). ^†^Significantly different from the corresponding data in Veh-treated neurons (*P* < 0.05). **(B)** The effect of TNFα treatment on the [Ca^2+^]_i_ responses to increasing concentrations of Cap in neurons isolated from WT and TNF^−/−^ mice. In the WT+TNFα group, 76 neurons were also included as part of the data presented in **(A)**. Data are mean ± SEM. ^*^Significantly different from the corresponding response to 30 nM capsaicin (*P* < 0.05). ^†^Significantly different from the corresponding data in TNF^−/−^ neurons (*P* < 0.05).

A total of 15 WT and 7 TNF^−/−^ mice were used in this study. Only the cultured jugular-nodose ganglia neurons innervating the lung structure identified by the DiI fluorescence were selected for our study. *Study 1:* In these isolated pulmonary sensory neurons, Cap evoked a rapid and transient increase in the [Ca^2+^]_i_ in a concentration-dependent manner (e.g., Figure 6). In the WT+TNFα neurons incubated with TNFα (50 ng/ml) for ~24 h, the Cap-evoked increase in [Ca^2+^]_i_ was significantly greater than that in the WT+Veh neurons (Figure 7A). The difference in the response of [Ca^2+^]_i_ between these two groups of neurons was greater at higher concentrations of Cap (e.g., 100 and 300 nM; Figure 7A); Δ[Ca^2+^]_i_ evoked by Cap (300 nM, 30 s) was 134.5 ± 44.6 nM in WT+TNFα neurons (*n* = 114), which was significantly greater than that in WT+Veh neurons (47.1 ± 9.1 nM, *n* = 138; *P* < 0.05). There was no difference in Δ[Ca^2+^]_i_ in response to the lowest dose of Cap (30 nM) between the two groups. *Study 2:* When the responses of [Ca^2+^]_i_ to increasing concentrations of Cap (30, 100 and 300 nM) were compared between pulmonary sensory neurons isolated from WT mice and TNF^−/−^mice after both groups of neurons were incubated with TNFα (50 ng/ml) for ~24 h, the Cap-evoked response was markedly attenuated in the TNF^−/−^ neurons (e.g., Figure 6); this difference in the response of [Ca^2+^]_i_ between these two groups of neurons was greater at the higher concentrations of Cap (300 nM; Figure 7B): Δ[Ca^2+^]_i_ = 74.8 ± 12.3 nM (*n* = 191) in the TNF^−/−^+TNFα group, and 143.2 ± 46.0 nM (*n* = 111; *P* < 0.05) in the WT+TNFα group. In this WT+TNFα group, 76 neurons were also included in the data presented in Figure 7A.

## Discussion

Results of this study showed that instillation of TNFα into the lung 24–48 h earlier induced a distinct increase in the sensitivity of vagal pulmonary C-fibers to Cap in anesthetized mice. More interestingly, the TNFα pretreatment also clearly elevated the sensitivity of silent RARs to Cap; in stark contrast, silent RARs exhibited no or very little Cap sensitivity in the Veh-treated mice. Furthermore, the increased sensitivity to Cap was also present in the isolated pulmonary sensory neurons, indicating that the sensitizing effect is mediated primarily through a direct action of TNFα on these neurons. The fact that this effect was attenuated in TNF^−/−^ neurons further suggests the involvement of TNF receptors.

The afferent properties and functions of the three major types of vagal bronchopulmonary sensory receptors have been described extensively in several animal species including dog, cat, rabbit, guinea pig, and rat (Coleridge and Coleridge, 1984; Lee and Yu, 2014; Mazzone and Undem, 2016). In more recent years, valuable information about the characterization and classifications of these vagal airway afferents in mice have been reported (Kollarik and Undem, 2004; Zhang et al., 2006; Lin et al., 2011), which have paved the road for realizing the promising potential of various transgenic and knockout mouse models in advancing our knowledge about the physiological function of airway sensory receptors (Nassenstein et al., 2010; Nonomura et al., 2017). Results obtained in this study from the control (Veh) mice were in general agreement with what have been reported by other investigators in this species; both SARs and phasic RARs are typical mechanoreceptors; they discharged phasically and synchronously with the respiratory cycles of the ventilator, and responded vigorously and consistently to lung inflation and/or deflation. The “intermediate receptors” described by previous investigators Zhang et al. (2006) were also categorized as SARs due to the standard AI criterion adopted in our study. Intravenous bolus injection of Cap did not evoke any significant stimulatory effect on SARs and phasic RARs in either Veh- or TNFα-treated mice. In sharp contrast, the same Cap challenge evoked a pronounced stimulatory effect on the silent RARs in the TNFα-treated mice. Questions regarding the reflex responses elicited by activation of these silent RARs still remain to be answered, as a distinct TRPV1 sensitivity emerged in these airway afferents after the TNFα treatment. The firing behavior of these silent RARs exhibited certain features resembling the “high-threshold Aδ vagal afferents” described by Lin et al. (2011); for example, they displayed a very mild sensitivity to Cap and other chemical stimulants of C-fibers (e.g., sulfur dioxide; data not shown), and did not exhibit phasic discharge during mechanical ventilation. In addition, we cannot rule out the possibility that these RARs are the cough receptors that have been identified in other species by previous investigators (Widdicombe, 1954; Canning et al., 2004). These silent RARs appear to be less frequently found and may represent a minority subgroup of the RARs though we did not attempt to determine their percentage distribution in this study.

Vagal C-fiber afferents represent the major type of the sensory nerves arising from the lung and airways (Jammes et al., 1982) and innervate the entire respiratory tract in various mammalian species including mice (Coleridge and Coleridge, 1984; Watanabe et al., 2006; Nassenstein et al., 2010; Lee and Yu, 2014). The afferent activity generated by these C-fiber endings plays a significant role in eliciting the pulmonary defense reflexes in both healthy and disease conditions (Coleridge and Coleridge, 1984; Lee and Yu, 2014). When these afferent endings are activated by either inhaled chemical irritants or endogenous inflammatory mediators, they can evoke respiratory sensations such as airway irritation, urge to cough, and dyspnea (Burki and Lee, 2010; Lee and Yu, 2014). In addition, it elicits powerful reflex responses including cough, airway smooth muscle contraction, bronchial vasodilation, and mucous hypersecretion (Coleridge and Coleridge, 1984; Lee and Pisarri, 2001); Intense and/or sustained stimulation of these afferents causes “neurogenic inflammation” in the tracheobronchial tree: extravasation of macromolecules and inflammatory cell chemotaxis. These airway responses are known to be generated through activations of both the cholinergic reflex pathways and the local “axonal reflex;” the latter involves the release of several bioactive tachykinins and calcitonin gene-related peptides from these sensory endings (Baluk et al., 1992; De Swert and Joos, 2006). Although we did not measure these responses in this study, it seems plausible and logic to postulate that when the C-fiber afferent sensitivity is elevated by an increase in the endogenously released TNFα, these airway reflex responses to a given stimulus will be intensified.

An important role of TNFα in the pathogenesis of allergic inflammatory diseases such as asthma has been extensively documented (Thomas, 2001; Howarth et al., 2005; Berry et al., 2006; Heffler et al., 2007; Brightling et al., 2008). TNFα was detected in bronchoalveolar lavage fluid, exhaled breath condensate and sputum of asthmatic patients during acute exacerbation or after antigen inhalation challenge in these patients (Keatings et al., 1997; Matsunaga et al., 2006). TNFα is released from a variety of cell types in the airways, such as mast cells and macrophages, via the immunoglobulin E-dependent mechanism (Gosset et al., 1992; Cembrzynska-Nowak et al., 1993; Thomas, 2001; Brightling et al., 2008). Once released, TNFα can exert multiple potent effects on a number of effector cells and induce the inflammatory reaction in the airways. Inhalation of aerosolized TNFα can also induce airway hyperresponsiveness accompanied by airway inflammation in healthy human volunteers (Thomas et al., 1995). Results of this study suggest that our finding of the TNFα-induced hypersensitivity of bronchopulmonary C-fiber afferents may be involved, at least in part, in the development of the airway hyperresponsiveness.

The mechanism(s) underlying the potentiating effect of TNFα on these vagal bronchopulmonary sensory nerves in this study is not yet fully understood. TNFα is known to exert its biological effects via a direct action on TNFR1 and TNFR2 that are present on a wide variety of cell types in the airways (Heffler et al., 2007). Upon activation by TNFα, these TNF receptors can actuate several signaling pathways that are involved in a wide range of immunological responses and inflammatory reactions (Brockhaus et al., 1990; Tartaglia and Goeddel, 1992; Baud and Karin, 2001; Utreras et al., 2009). TNFα is initially produced as a membrane-anchored precursor protein, and subsequently cleaved into free proteins, which form biologically active homotrimers and interact with these TNFRs. TNFα can act in a paracrine manner on epithelial cells in promoting the transmigration of leukocytes via a TNFR1-dependent signaling pathway (Morton et al., 2016). Activation of TNFR1 is also known to activate nuclear factor-kappa B (Furukawa and Mattson, 1998) and mitogen activated protein kinases (MAPK) pathways, including the extracellular signal-regulated kinase (ERK; Barbin et al., 2001). Hensellek and coworkers (Hensellek et al., 2007) have demonstrated that exposure of DRG neurons to TNFα for 24–48 h significantly increased the proportion of DRG neurons expressing TRPV1 receptor-like immunoreactivity via TNFR1 and the ERK activation. Furthermore, it has been shown that activation of p38 MAPK pathway increased TRPV1 expression in peripheral nociceptor neurons in a transcription-independent manner (Ji et al., 2002). Indeed, our immunohistochemical study has previously demonstrated the presence of both TNFR1 and TNFR2 on the cell membrane of rat vagal pulmonary sensory neurons (Hu et al., 2010). Results obtained in the present study showing that the sensitizing effect of TNFα was attenuated in isolated TNF^−/−^ neurons lend an additional support to this hypothesis. In addition, activation of TNFRs can also cause a chemotactic action, upregulate the leukocyte-endothelial cell adhesive molecules, enhance the production of Th2 cytokines and cause infiltration and degranulation of these inflammatory cells in the airways (Kips et al., 1992; Bradding et al., 1994; Thomas et al., 1995; Thomas, 2001; Nakae et al., 2007). Thus, these actions of TNFα can evoke releases of other inflammatory mediators, and some of these autacoids such as histamine, leukotrienes, and thromboxanes (Lin et al., 2013) are known to exert pronounced sensitizing effects on vagal bronchopulmonary C-fiber afferents (Lee and Yu, 2014).

Another interesting observation should be further elaborated. When the silent RARs were activated by Cap injection in TNFα-treated mice, the evoked fiber activity was frequently phasic and synchronous with the inspiratory cycles of the ventilator (e.g., Figure 3C); this phasic discharge pattern was distinctly different from that in C-fiber response to Cap (e.g., Figure 1). We don't believe that the phasic activity was related to bronchoconstriction because the discharge occurred immediately (in 1–2 s) after the Cap injection when there was no detectable change in peak tracheal (e.g., Figure 3C). Furthermore, the TNFα treatment did not increase the response to lung inflation in silent RARs. Interestingly, in the TNFα-treated mice the phasic activity of these RARs was only found during the short interval immediately following the Cap injection, similar to the duration and time course of the C-fiber response. We suggest that a possible mechanism may involve the expression of TRPV1 on the sensory terminals of the silent RARs induced by the TNFα treatment. Activation of TRPV1 by the Cap injection may then trigger Ca^2+^ influx and lead to subthreshold depolarization of the terminal membrane. In addition, the presence of Cap can shift the TRPV1 channel activation curve (open probability vs. voltage) to a physiologically relevant voltage range with a relatively small gating charge (Voets et al., 2004). Both of these actions generated by Cap can increase the membrane excitability. Thus, a below-threshold stimulus to the mechanosensitive channels, such as a lung expansion during the inspiratory cycle of the ventilator, may trigger the firing of action potentials at the sensory terminals of these silent RARs.

In conclusion, this study demonstrated that the intra-tracheal instillation of TNFα 1–2 days earlier significantly enhanced the sensitivity of pulmonary C-fibers to Cap and upregulated the TRPV1 sensitivity in a subgroup of RARs. However, the underlying mechanism of the TNFα-induced change in the TRPV1 sensitivity of these afferents is still not yet fully understood. As this study has established the evidence of this sensitizing effect in mice, the potential involvements of certain TNFR-mediated signaling pathways (discussed earlier) can be further explored in the transgenic and knockout mouse models. Considering the increasing evidence suggesting a possible involvement of the TRPV1 channel in the manifestation of a number of symptoms of airway hypersensitivity during inflammatory reaction, a possible impact of the sensitizing effects of TNFα on these airway afferents and their regulation of overall cardiopulmonary functions in allergic airway inflammatory diseases certainly merits further investigations.

## Author contributions

All three authors, RL, QG, and LL, are responsible for designing the study, performing experiments, collecting, analyzing, and interpretation of the data, and preparation of the manuscript.

### Conflict of interest statement

The authors declare that the research was conducted in the absence of any commercial or financial relationships that could be construed as a potential conflict of interest.
